# Systematic Single-Cell Analysis of *Pichia pastoris* Reveals Secretory Capacity Limits Productivity

**DOI:** 10.1371/journal.pone.0037915

**Published:** 2012-06-07

**Authors:** Kerry Routenberg Love, Timothy J. Politano, Vasiliki Panagiotou, Bo Jiang, Terrance A. Stadheim, J. Christopher Love

**Affiliations:** 1 Department of Chemical Engineering, Koch Institute for Integrative Cancer Research, Massachusetts Institute of Technology, Cambridge, Massachusetts, United States of America; 2 GlycoFi, a wholly-owned subsidiary of Merck and Co., Lebanon, New Hampshire, United States of America; University of Illinois at Urbana-Champaign, United States of America

## Abstract

Biopharmaceuticals represent the fastest growing sector of the global pharmaceutical industry. Cost-efficient production of these biologic drugs requires a robust host organism for generating high titers of protein during fermentation. Understanding key cellular processes that limit protein production and secretion is, therefore, essential for rational strain engineering. Here, with single-cell resolution, we systematically analysed the productivity of a series of *Pichia pastoris* strains that produce different proteins both constitutively and inducibly. We characterized each strain by qPCR, RT-qPCR, microengraving, and imaging cytometry. We then developed a simple mathematical model describing the flux of folded protein through the ER. This combination of single-cell measurements and computational modelling shows that protein trafficking through the secretory machinery is often the rate-limiting step in single-cell production, and strategies to enhance the overall capacity of protein secretion within hosts for the production of heterologous proteins may improve productivity.

## Introduction

More than 240 monoclonal antibody products are currently in clinical trials [1], and protein-based products are expected to constitute four of the five top selling drugs by 2013 [2]. While mammalian cells and *Escherichia coli* are the main production hosts for biopharmaceutical manufacturing, yeast cells have also proved to be useful hosts owing to their stability and capability to secrete complex proteins. *Pichia pastoris* is a methylotrophic yeast that is a widely used host for heterologous protein expression [3], [4]. Engineering these organisms has also generated strains capable of secreting monoclonal antibodies with homogeneous human *N*-linked glycans in excess of 1 g/L [5], [6]. A potential protein therapy for cancer and rheumatoid arthritis (α-IL-6 monoclonal antibody) produced in *P. pastoris* is currently in Phase II clinical trials (http://www.alderbio.com/11/PIPELINE/). Despite the increasing importance of *P. pastoris* in biomanufacturing, its productivity per culture still lags the state-of-the-art mammalian cell lines. The yield of protein produced by fermentation is one of the most significant factors in determining both the cost of biotherapy production [7] and ultimately, can impact global access to therapies for patients. A key goal of any bioprocess development, therefore, is to maximize protein production and secretion from the host cells while maintaining product quality and consistency.

One route to optimize productivity is through rational strain engineering. Engineering promoters [8], [9] or over-expressing either transcription factors [10] or specific proteins in the secretory pathway [11], [12] in *P. pastoris* has led to moderate increases in productivity on a case-by-case basis, but cultivation titers have been reported to vary dramatically with protein type and complexity. For example, non-glycosylated, monomeric proteins, such as human serum albumin (HSA), can be produced in fermentation with yields up to 10 g/L [13]. Secretion of more complex proteins in *P. Pastoris*, including multimeric structures with post-translational modifications, are challenging, however, to produce in excess of 1 g/L [5].

Population-based analysis of the genome [14], [15], [16], transcriptome [17], [18], [19], [20], and proteome [21] has identified certain genes that may further increase productivity, but a general understanding of the most influential factors that affect the yield of secreted proteins from *P. pastoris* has not yet developed. Non-genetic factors also introduce substantial variability among cells that further influences both production and secretion of proteins. Recent reports of significant intraclonal variation in protein secretion by both CHO cells [22] and *P. pastoris* [23] demonstrate that epigenetic factors can strongly influence the distribution in productivity within a culture. A systematic characterization of the dynamics and variation in the production and secretion of proteins at the single-cell level is needed to assess the diversity among a population of cells and ultimately, to establish a conceptual framework for informing strain engineering.

Here, we present a methodical investigation to determine how the nature and complexity of heterologous proteins impacts the efficiency with which they are secreted by *P. pastoris* at the cellular level. Using tools we have previously developed to examine the secretions from single cells, we show directly that the key bottleneck in protein secretion is the capacity of the secretory machinery to transport folded protein out of the endoplasmic reticulum (ER) and beyond. We then describe a simple computational model for the flux of folded protein through the ER based on a series of ordinary differential equations that further supports these experimental observations and provides mechanistic insights to the rate-limiting steps in this process. Furthermore, the resulting understanding of how the nature of the protein produced intersects with intrinsic limitations on secretory flux resolves many of the variations reported for protein secretion in yeasts.

## Results

### Strain construction and characterization

A series of yeast strains that each secreted one of three different proteins of increasing folding complexity was generated. We selected enhanced green fluorescent protein (eGFP), which is known to mature rapidly (∼30 min) and spontaneously [24], to enable monitoring of intracellular, folded protein in relation to secreted, folded protein. For comparison, we also chose to examine both glycosylated and aglycosylated versions of a human Fc fragment, a dimeric protein that requires foldases and chaperones for proper folding [25]. To control for variations in coarse transcriptional activities, all strains used the same locus (GAPDH) for insertion of the gene of interest. For each strain, we also determined the number of copies of the inserted gene by qPCR and the relative expression of the gene at steady-state during cultivation by RT-qPCR (Table 1).

**Table 1:** *Pichia pastoris* strains generated for systematic investigation of the relationship between protein complexity, gene dosage, relative expression and secretion.

Strain Name^a^	Secreted protein	Gene copy^b^ number	Relative mRNA^c^ Expression
pGAPαeGFP1	eGFP	1	2.1
pGAPαeGFP2	eGFP	3	12.7
pGAPαeGFP3	eGFP	≥4	19.4
pGAPαGFc1	Glycosylated Fc	1	1
pGAPαGFc2	Glycosylated Fc	2	1.4
pGAPαGFc3	Glycosylated Fc	≥6	11.3
pGAPαAgFc1	Aglycosylated Fc	1	5.1
pGAPαAgFc2	Aglycosylated Fc	2	5.4
pAOXαeGFP1	eGFP	2	16.4
pAOXαeGFP2	eGFP	5	19.8
pAOXαGFc1	Glycosylated Fc	2	10.9
pAOXαGFc2	Glycosylated Fc	5	8.9
pAOXαAgFc1	Aglycosylated Fc	2	5.1
pAOXαAgFc1	Aglycosylated Fc	4	22.2

a Strain names indicate promoter used.

b Determined by qPCR using an absolute quantification of transcript copy number.

c Determined by RT-qPCR using an absolute quantification of transcript copy number and the expression of actin as a control.

**Figure 1:**
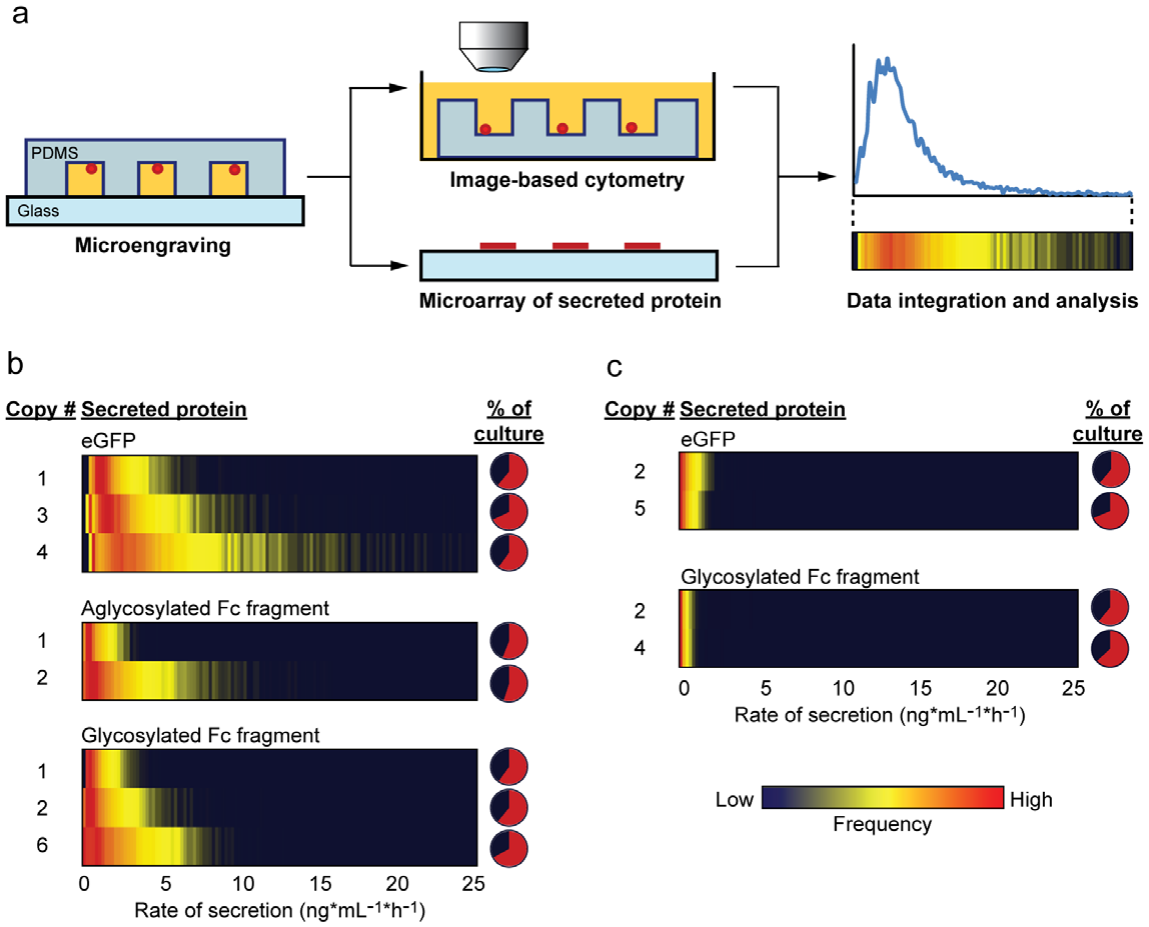
Single-cell analysis of *P. pastoris* secreting heterologous proteins. (A) Schematic illustration of process for measuring the distributions in rates of secretion of heterologous proteins by single cells. Yeast cells cultivated by shake-flask fermentation for ∼12–24 h at 25°C were deposited onto an array of microwells at a density of ∼1 cell per well. Microengraving was performed to create a protein microarray comprising the secreted proteins captured from occupants of each individual well. Imaging cytometry was performed to determine number of cells per well. Integration of the data yielded distributions in rates of secretion for single cells; the distributions are represented as heatmaps where the gradient in color (blue to yellow to red) indicates the relative percentage of cells producing at a specific rate. (B) and (C) Heatmap representations of the distributions of single-cell rates of secretion as a function of gene copy number obtained by microengraving for proteins produced using either (B) pGAPDH or (C) pAOX1 promoter. Data shown are representative of at least three independent measurements. The threshold for secretion was determined by the background median fluorescence intensity of each individual protein microarray+2σ. Pie charts indicate average percentages of secreting cells for each strain (red).

We then used microengraving to monitor secretions quantitatively from thousands of individual cells within each strain (Figure 1A). Microengraving is a soft-lithographic technique for the high-throughput analysis of secreted products from single cells [26], [27], including *P. pastoris* [23]. The method reveals the percentage of secreting cells (similar to an enzyme-linked immunospot assay), as well as a measure of the average rate of secretion. Single-cell analysis of strains secreting proteins under transcriptional control of a constitutive promoter, pGAPDH, following shake-flask cultivation showed that protein complexity modestly affected the rate of protein secretion, but not the percentage of secreting cells within the population (Figure 1B). Under this promoter, single cells secreted eGFP slightly faster (1.4±0.2 ng*mL^−1^*h^−1^ average median rate for the single-copy strain) than either the glycosylated (0.9±0.3 ng*mL^−1^*h^−1^) or aglycosylated Fc fragment (0.7±0.2 ng*mL^−1^*h^−1^), which were secreted at similar rates. Single-cell rates of secretion increased linearly (R^2^ = 0.83) with gene expression for all strains assayed (Figure S1), indicating that when proteins are produced using a constitutive promoter, the cellular capacity for secretion does not fully saturate, as expected [28].

Only 65±10% of cells from any culture analyzed for these strains actively secreted detectable quantities of protein during our assays (typical limit of detection was between 0.05 and 0.2 ng*mL^−1^*h^−1^). We have previously determined that these “off” cells are neither dead nor geriatric [23]. Moreover, these cells are not metabolic mutants: measures of internal eGFP by in-well imaging cytometry revealed >97% of all single cells had detectable levels of internal protein, and the percentages of secreting cells were identical when grown for conditional exclusion of petite colony mutants (data not shown). Confocal microscopy images showed eGFP-secreting cells had a distinct distribution of the protein within an intracellular compartment close to the nucleus, consistent with localization in the ER and Golgi; by comparison, a strain producing eGFP that was not targeted for secretion had distributed protein uniformly throughout the cytoplasm (Figure S2). All cells assayed from a secreting clonal population, therefore, competently produce folded protein in the ER, but only a fraction of these cells actively secretes it at any given time.

Increasing the flux of protein into the secretory pathway using the strong and tightly regulated methanol-inducible promoter from the alcohol oxidase 1 gene (pAOX1) of this organism to promote transcription had a dramatic effect on the single-cell rates of protein secretion for all three proteins (Figure 1C). Their median rates of secretion were reduced 2–4 fold relative to their rates under pGAPDH for strains with low copy numbers of genes. Interestingly, the secretion rate for aglycosylated Fc was undetectable within the sampling period (60 min), suggesting slow rates of release below our single-cell limits of detection. We further confirmed that these strains did produce much lower titers of protein than the corresponding pGAPDH-strains by both SDS-PAGE and ELISA of culture supernatants (data not shown). This outcome may result from decreased levels of folded aglycosylated Fc available for export from the ER relative to the glycosylated Fc, since interactions with essential folding chaperones are promoted by glycosylation [29]. Increasing the copy number of genes under transcriptional control of pAOX1 further decreased rates of secretion for all proteins, consistent with literature reports of similar findings [11], [30]. The frequencies of secreting single cells were, however, similar to those observed under pGAPDH (60±10%). Non-secreting cells were neither dead nor incapable of making and folding proteins—98% of cells producing eGFP under pAOX1 had internal eGFP. Together, these results indicate that enhanced expression of proteins can negatively impact the median productivity of individual cells.

Surprisingly, however, expression levels of mRNA for genes transcribed using pAOX1 were not substantially more than that observed for high-copy strains of the same genes using pGAPDH (Table 1). This result may indicate degradation of mRNA transcript following induction of the unfolded protein response (UPR) when using pAOX1, or could also indicate that pAOX1 is not significantly more active than pGAPDH. We note that the observed relative expression levels are not likely due to poor culture induction since distributions of single-cell rates of secretion and cell-corrected protein titers are similar for glycosylated Fc secretion when cells are cultivated either in shake flask or in fed-batch fermentation (Figure S3).

### Modeling population distributions of secretion

**Figure 2:**
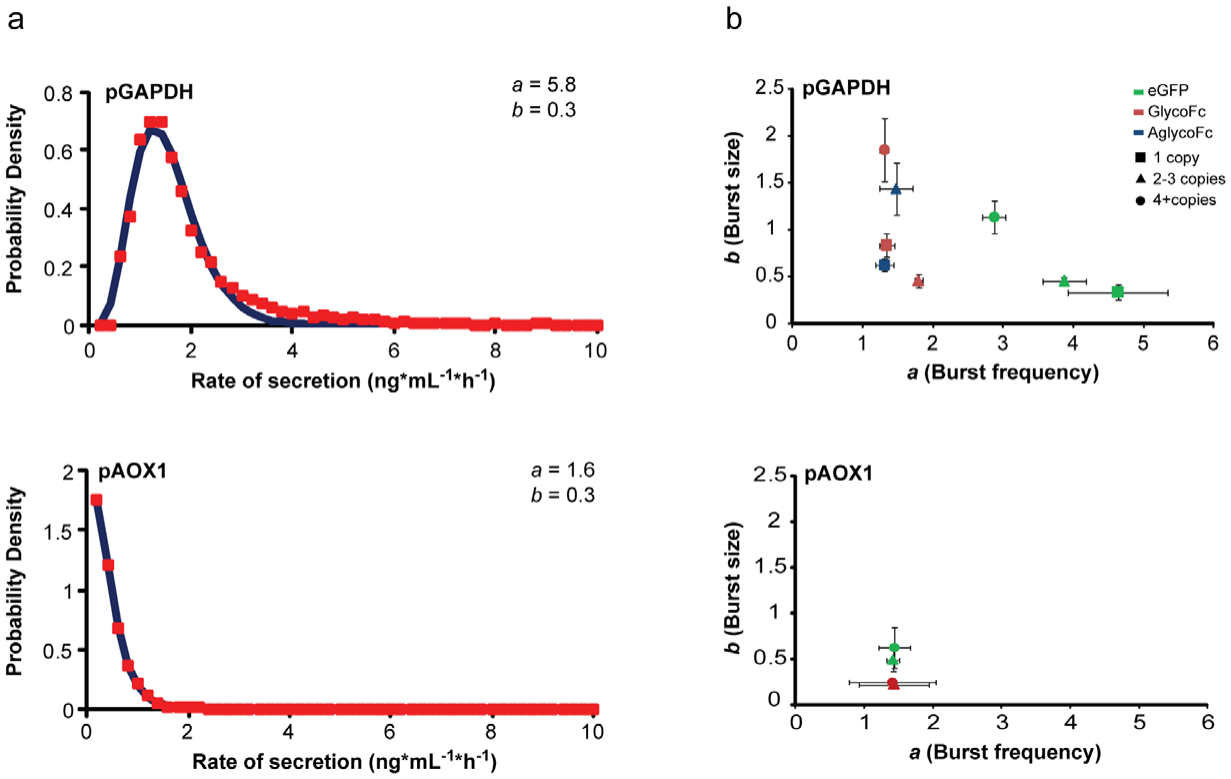
Analysis of steady-state distributions of rates of secretion. (A) Distributions of rates of secretion of eGFP for (Top) a clone with a single copy of eGFP under transcriptional control of pGAPDH, and (Bottom) a clone with two copies of eGFP under transcriptional control of pAOX1. Red squares indicate binned single-cell secretion events following microengraving with each clone. Blue lines show the best fits using Eq. (1). Values for *a* and *b* are shown. (B) Relationship between *a* (burst frequency) and *b* (burst size) for proteins expressed using either pGAPDH (top) or pAOX1 (bottom) as a function of gene copy number and complexity. Clones secreting eGFP (green), clones secreting aglycosylated Fc fragment (blue) and clones secreting glycosylated Fc fragment (red) are shown for a single gene copy (squares), 2–3 gene copies (triangles) and 4 or more gene copies (circles). Error bars represent S.E.M. for each clone from at least three separate measurements.

Our experiments showed that the rates of protein secretion by individual cells exhibited significant heterogeneity when transcription was mediated by either pGAPDH or pAOX1. Assuming that the release of secreted proteins from cells is a Poisson process, we modeled the steady-state distributions for all strains (Figure 2A) using a probability density function of the gamma distribution (Eq. 1):
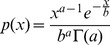
(1)where the probability of secreting protein at a given rate *(p(x))* depends on parameters *a*, the average rate of secretion events, and *b*, the average size of those events (proportional to the number of molecules released) [31]. This analysis for all strains indicated that variations in secretion depend on both the complexity and magnitude of gene expression of a protein (Figure 2B). As gene expression increases for strains using pGAPDH, the frequency of secretion events decreases, but the size of any given burst increases. This inverse relationship between *a* and *b* suggests that either the volumes of the vesicles transporting proteins or the total intrinsic capacity of the cell for protein export must be fixed—that is, there is a discrete set of components (membranes, proteins, etc.) available for shuttling protein from the ER through the Golgi apparatus to the cell surface. The export of protein out of the ER appears to be particularly burdened under pAOX1. Fitted distributions describing single-cell secretion rates for pAOX1 strains indicate both burst frequency and burst size both remain low, perhaps indicating inefficient recycling of protein export machinery in the presence of excess protein cargo.

### Export of proteins by secretion is rate-limiting

**Figure 3:**
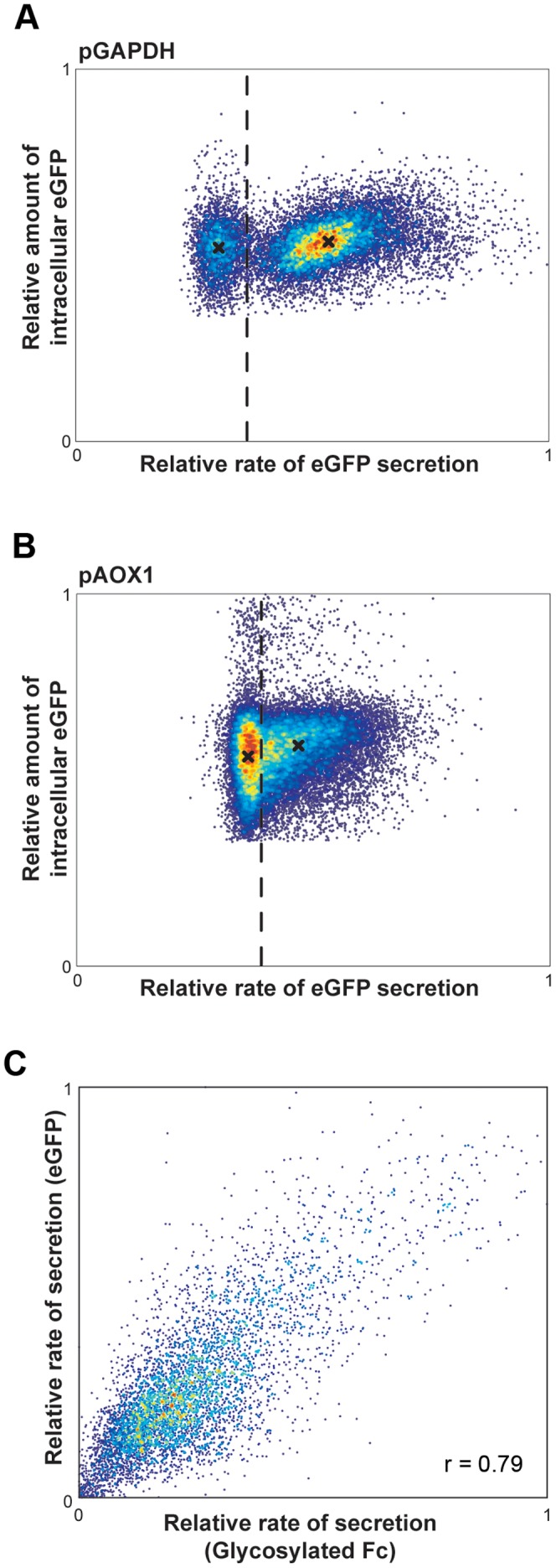
Characterization of relationships between intracellular and secreted proteins for single cells of *P. pastoris*. Density plots of the relative rates of eGFP secretion by single cells analyzed by microengraving with respect to the relative amount of intracellular eGFP determined by fluorescence microscopy for clones containing (A) one eGFP gene copy under pGAPDH or (B) two eGFP gene copies under pAOX1. Dashed line indicates the limit of detection for secreted eGFP in microengraving (background+2σ). The median amounts of internal eGFP for cells above and below this limit of detection are marked (X) and are significantly different (Mann-Whitney test, p≪0.001 for both pGAPDH and pAOX strains). (C) Density plot of the relative rates of secretion analyzed by microengraving for the glycosylated Fc fragment and eGFP produced simultaneously in single cells at two different loci using pGAPDH. Pearson's correlation coefficient for secretion of these two proteins as shown is 0.79.

Strains producing eGFP under either pGAPDH or pAOX1 showed that nearly all cells produced folded protein, but only a subset was secreting it. Further comparison of the relative rates of secretion of eGFP to intracellular quantities in individual cells showed two distinct, co-existing subpopulations: one that secretes eGFP at detectable levels and another that is non-secreting (Figure 3A and 3B; File S1). Interestingly, both populations have similar amounts of folded intracellular eGFP, with the secreting population containing slightly more protein (p<0.001). The population that actively secreted protein did so at a rate moderately proportional to the amount of intracellular eGFP (pGAPDH, r = 0.18; pAOX1, r = 0.06). Induction of UPR by treating cells with the strong reducing agent dithiothreitol (DTT), known to specifically affect protein folding within the secretory pathway [32], led to a six-fold reduction in the median rate of eGFP secretion (data not shown). Nonetheless, the correlation between accumulation of folded protein and secretion in the actively secreting population was maintained (pGAPDH, r = 0.12). Furthermore, the non-secreting populations of both the untreated and DTT-treated cultures had similar amounts of intracellular eGFP, indicating that the UPR itself does not lead to an excess accumulation of folded protein inside the ER. The proportionality between intracellular levels of eGFP and rates of secretion—even under conditions of induced UPR—suggest that the flux of folded proteins out of the ER, and subsequently through the secretory pathway, is a rate-limiting process for productive export.

### Rates of secretion and degradation determine steady-state distributions of folded protein in the ER

**Figure 4:**
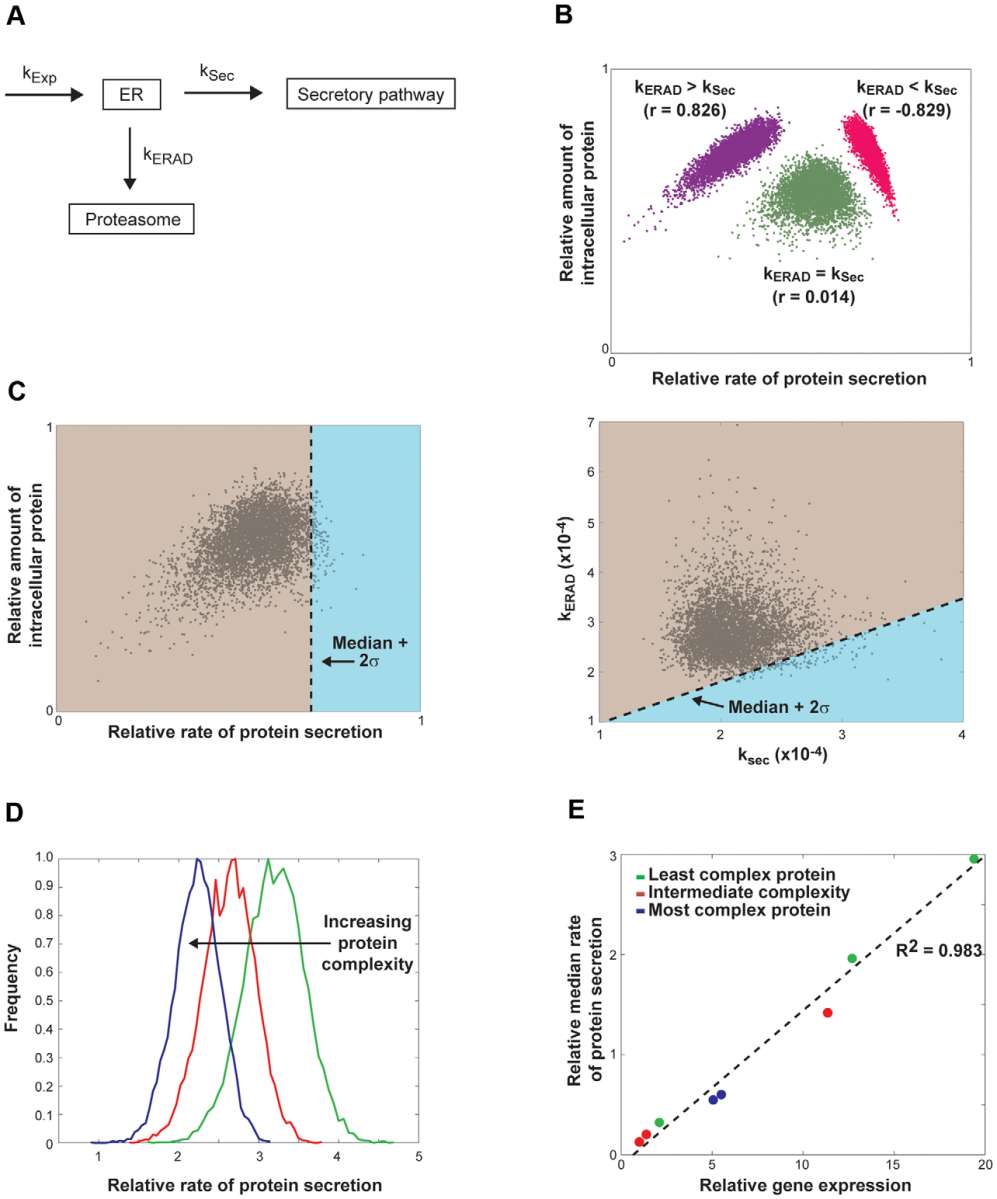
Model for steady-state distribution of protein trafficking through the ER in *P. pastoris*. (A) Schematic model for protein secretion that includes flux from the ER via both protein export and degradation. (B) Density plot of the relative rates of protein secretion by single cells compared to the relative amount of intracellular protein calculated with the kinetic model described in equations 2–4 under different relative median rates: k_ERAD_>k_sec_ (purple, r = 0.826); k_ERAD_ = k_sec_ (green, r = 0.014); and k_ERAD_<k_sec_ (pink, r = −0.829) (n = 5,000 for each group; Pearson's correlation coefficient for protein production and secretion). (C) Density plot of the relative rates of protein secretion by single cells against the relative amount of intracellular protein for a representative model data set where median k_ERAD_>k_sec_ (left panel). Pearson's correlation coefficient for protein production and secretion in this population is 0.391. Blue shading indicates cells with rates of protein secretion greater than the median rate+2σ (high secretors). These data were replotted as a function of their rate parameters for secretion and degradation; units shown are s^−1^ (right panel). The median t_sec_ was 80 min and median t_ERAD_ was 60 min, with a standard deviation of 10 min for each. (D) Distributions of the relative rates of protein secretion for model populations of cells producing proteins of low (green), intermediate (red), and high complexity (blue). The median t_sec_ value was scaled by a multiplicative constant between 1 and 2 in order to reflect the additional time required to process proteins of higher complexity. (E) Plot of relative gene expression against the median rates of secretion for populations of cells generated using the kinetic model with varying levels of gene expression and protein complexity. Data were fit by linear regression (R^2^ = 0.983). k_exp_ was multiplied by the relative mRNA expression level in each strain (Table 1), and t_sec_ was scaled as noted in (D) for glycosylated Fc (medium complexity) and aglycosylated Fc (high complexity).

We next sought to develop a simple mechanistic model from first principles to understand how distinct subpopulations of cells with varied rates of secretion could arise. The flux of proteins through the ER is determined by the rates at which proteins transfer into the ER, and then out of the ER either by entering the secretory pathway or by being shuttled to the proteasome via ER-associated degradation (ERAD) [33]. We generated a mathematical model (Eqs. 2–4) to describe the steady-state distribution of folded proteins retained in the ER (Figure 4a):
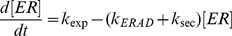
(2)

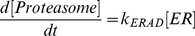
(3)

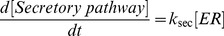
(4)where [ER] represents the concentration of folded protein present in the ER and k_exp_, k_ERAD_, and k_sec_ are the rate constants for protein flux into the ER, out of the ER to the proteasome, and out through the secretory pathway, respectively.

We hypothesized that protein transcription and translation were not significant determinants for the overall rate of secretion. A typical protein is transcribed and translated within less than 10 minutes per step [34], [35], while transit of proteins through the ER and Golgi typically requires 40 to 120 minutes per step [28]. It also has been demonstrated that factors extrinsic (downstream) to expression of a gene dominate the variation in the intracellular quantity of folded proteins that are highly abundant [36]. Given these reports, we tested our assumption by creating a strain concurrently secreting two proteins of different folding complexity (eGFP and glycosylated Fc) transcribed from different loci within the same cell (Figure 3C). If the rate-limiting step for secretion were strongly influenced by the rates of gene transcription or translation for each product, we would expect no correlation between the rates of secretion for these two proteins since the behaviors observed for individual cells would depend on the stochastic bursts of transcription/translation associated with each product independently. Instead, we observed that the secretion of both eGFP and the Fc fragment by single cells was highly correlated (r = 0.79–0.9) in spite of the expression of these proteins from different loci [37]. This correlation supports a kinetic model in which the transit of folded proteins through the ER and Golgi is the rate-limiting process, while the distribution observed among cells' combined rates of secretion for both proteins suggests that additional extrinsic factors affect the capacity of any given cell to secrete proteins.

Using our mathematical model, we then further examined the relationship between the rate of protein secretion and the amount of intracellular protein under a variety of conditions. We simulated the steady-state secretion from cells by initializing the ODEs above (Eqs. 2–4) with varying values of k_exp_, k_ERAD_, and k_sec_. The ODEs were then solved to achieve a steady-state condition over time for a cell starting with no protein in the ER, proteasome, and secretory pathway (as detailed in the Methods). Once the system obtained steady-state, the rate of secretion and quantity of protein in the ER were recorded. To model data for a distribution of cells, this simulation was repeated 5,000 to 10,000 times, allowing each iteration to select kinetic parameters from a Gaussian distribution centered around the initial values used to establish the relative rates of secretion and degradation (1/k_sec_ and 1/k_ERAD_, respectively).

Global changes to the relative rates of protein degradation and protein secretion in the model dramatically affected the dependence between the amount of intracellular protein and the relative rate of protein secretion (Figure 4B). When the median rate of protein degradation through ERAD in the population exceeded that of protein secretion, there was a positive linear correlation between the rate of secretion and the amount of intracellular protein. This outcome was consistent with our experimental data for the production and secretion of eGFP (Figure 3). Indeed, UPR, and by extension ERAD, are known to be constitutively active in *P. pastoris* [10]. Decreasing the median rate of protein degradation relative to protein secretion abolished this positive correlation, and once the median rate of protein secretion exceeded degradation, the correlation was negative.

The model also suggested, therefore, that the subpopulation of cells with the highest median rates of secretion within a distribution would have the highest proportional rate of secretion compared to degradation. For a modeled distribution of cells similar to our experimentally measured ones (k_ERAD_>k_sec_), the best predicted secretors (median+2σ) were those that exceeded a certain threshold ratio of k_sec_/k_ERAD_ (Figure 4C). These predictions together indicate that the relative rates for flux of protein out of the ER (via ERAD and via the secretory pathway) can strongly influence the relationship between intracellular contents and rates of secretion observed experimentally.

We then modified the model to include a scaling factor for k_sec_ to account for other biomolecular attributes that can impede the overall rates of a protein's progression to secretion (e.g., maturation time, post-translational modifications, or multimerization). Varying this parameter showed that the median rate of secretion decreased as the complexity of the proteins increased (Figure 4D), confirming another experimental observation (Figure 1B). Furthermore, our simple model for the steady-state distribution of proteins in the ER also confirmed our experimental observation that secretion scales linearly with gene expression among proteins with diverse complexity (Figure 4E and Figure S1).

## Discussion

Here, we report a systematic experimental and computational investigation into how the rate of secretion varies among individual cells relative to the complexity and expression level of heterologous recombinant proteins in the yeast *P. pastoris*. Modeling distributions of the rates of secretion exhibited by single cells showed that an inverse relationship exists between the frequency of secretion events and the quantity of protein in each event for proteins of varying complexity. This relationship indicates that there is a fixed capacity for protein secretion that is independent of the capability of a cell to fold a particular protein. Variations in secretion observed within a culture result, therefore, from heterogeneities in the capacities of individual cells to export proteins by secretion, rather than from their inability to prepare folded proteins for secretory export.

Constraining the flux of proteins out of the ER via the secretory pathway also implies that folded proteins accumulate in the ER, and further promotes the induction of the UPR (including ERAD) as the machinery available for exporting protein becomes saturated. Our simple model for the steady-state quantities of protein in the ER best agreed with our experimental observations when flux out of the ER via degradative processes increased. The excess of protein entering the secretory pathway when using active promoters like pAOX1, therefore, likely congest protein export machinery more rapidly than constitutive promoters, and subsequently causes high levels of ER stress [12], protein degradation [38] and reduced translation [39]. These responses may then further erode the median rate of protein secretion because less protein remains available for export relative to the quantities degraded.

**Figure 5:**
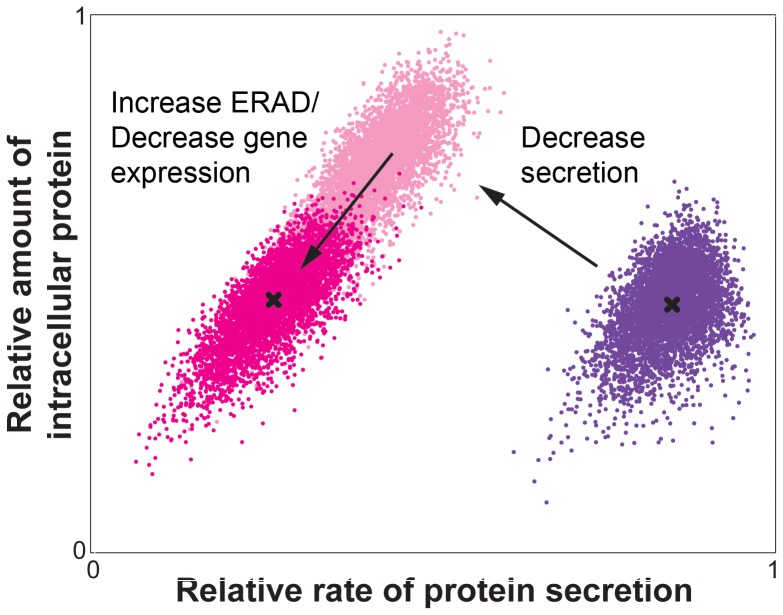
Effects of altering relative rates of secretion and degradation on modeled distribution of intracellular and secreted protein. Density plot of the relative rates of protein secretion by single cells against the relative amount of intracellular protein for model data sets under three conditions: 1) where median k_ERAD_≥k_sec_ (purple), 2) where median k_sec_≪k_sec_ in Condition 1 (light pink), and 3) where median k_sec_≪k_sec_ in Condition 1 and median k_ERAD_>k_ERAD_ and/or k_exp_<k_exp_ in Condition 1 (dark pink). The median amount of intracellular protein for populations in Conditions 1 and 3 are marked (X). The secretion-inhibited population (light pink) was generated by increasing t_sec_ to 400 min (standard deviation 40 min) while keeping all other parameters the same as the initial population (purple) derived from Figure 4C. The stress-induced population (dark pink) was generated by reducing k_exp_ or decreasing t_ERAD_ to obtain a similar median level of protein in the ER as the original population.

Our data here also indicates that secretion is a binary phenotype for individual cells: eGFP-producing cells co-exist as two distinct populations of cells with one actively secreting and one essentially “off” population (Figure 3). We previously reported that cells can switch dynamically between these two states of secretion [23]. Here, we have proposed a simple model to explain the steady-state distribution of folded proteins trafficking through the ER (Eqs. 2–4). This model provides mechanistic insight into how cells may transition between secreting and non-secreting states. Considering an initial population of secreting cells (purple, Figure 5) similar to those we observed experimentally (where the median rate of ERAD is greater than the median rate of secretion), it is expected that increasing stores of folded protein in the ER leads to the saturation of the available capacity for secretion and a subsequent decline in k_sec_. If k_ERAD_ is constant during this secretory decline, our model suggests that an accumulation of intracellular protein should occur, as incoming folded proteins are neither efficiently secreted from the ER nor degraded (light pink, Figure 5). A concomitant increase in k_ERAD_ and/or decrease in k_exp_ is required to reduce residual folded protein and recover a distribution of cells with an intracellular protein level that is similar to that in the secreting populations (dark pink, Figure 5). A consequence of this upregulated activity, however, is that the median rate of secretion also decreases. This outcome is comparable with our experimental results, where the median amount of protein inside the non-secreting populations of pGAPDH or pAOX1 eGFP-producing strains is similar to that found in secreting populations (Figure 3). In fact, non-secreting populations of cells in strains using either promoter contain statistically lower amounts of intracellular eGFP. This observation is consistent with a mechanism in which cells under stress switch to an “off” state of secretion wherein excess folded protein is depleted from the ER and the flux of incoming proteins slows prior to restarting secretion.

The observed “all-or-none” secretion phenotype is not consistent with the existence of an analog transition between secreting to non-secreting states, and implies that the cells use positive feedback to regulate these transitions [40]. Bistability in biological systems is now widely recognized [41], and the ability of a cell to efficiently transition from one state to another can confer a fitness advantage [42]. Maintaining a dynamic balance between exporting proteins to the Golgi and ERAD, which is essential for secretion [43], is likely a key mechanism for cell survival. Accumulation of protein within the ER can result in reticulophagy (ER-specific autophagy) [44] and ultimately, cell death [43]. Cells that have fully activated an ERAD-pathway shunt may, however, require time to regain previous levels of secretory function. This hypothesis is also consistent with our previous observations that at least several doubling times are necessary for single cells (or their progeny) to regain productivity [23]. Our simple model based on mass balance does not explicitly account for the sharp transitions observed, and it is likely that both a complex network of genes regulating secretion and epigenetic variations contribute to the cellular decision-making process when switching between states of secretion [45]. Understanding this interplay will be essential for a complete understanding of the dynamic processes linking secreting and non-secreting phenotypes.

Nonetheless, many of the variations reported in the literature regarding protein secretion in yeast can be accounted for by invoking a mechanism wherein an intrinsic capacity for flux through the ER into the secretory pathway limits the transit of protein, and subsequently ER stress increases as folded proteins accumulate in the ER. General activation of the UPR decreases expression and secretion for small proteins with few disulfide bonds [10]. This outcome is understandable in light of this study, since UPR involves upregulation of associated transcription factors like HAC1 that also trigger increased ERAD through an upregulation of other proteins [32], including ER degradation-enhancing mannosidase-like protein (EDEM) [33], a homolog of which exists in *P. pastoris* [14]. Indeed, ERAD-associated E3 ubiquitin-protein ligase HRD1 was observed to be upregulated in *P. pastoris* overexpressing Hac1 [18].

Activation of the UPR can be beneficial, however, when protein folding, rather than transit through the secretory pathway, becomes rate limiting. In addition to inducing increased ERAD, expression of HAC1 also upregulates certain foldases and chaperones in *P. pastoris*, such as protein disulfide isomerase (PDI) and binding immunoglobulin protein (BiP) [18]. Targeted upregulation of these factors has enhanced the production of complex proteins more than general activation of the UPR, since there is not an accompanying increase in degradation [11], [12]. Furthermore, a specific increase in the expression of calnexin, another ER-resident chaperone, promotes increased secretion of many proteins, regardless of protein complexity or post-translational modification [46]. These enhancements reduce the time required for protein folding of complex proteins, and improve productivity, since secretion is not yet rate limiting in these cases. Dramatic increases in the amount of protein present in the ER, either by copy number increase or promoter improvement, typically lead to decreased production [12], [30], as these changes likely overwhelm the secretory machinery. These outcomes together are consistent with our model in which secretory capacity is fixed, and folded proteins accumulate in the ER faster than they are secreted.

We posit that these results have three implications for improving the secretion of heterologous proteins. First, targeting components involved in transport from the ER to the Golgi and out of the cell, as well as developing new strategies to control ERAD and protein degradation, should increase fermentation titer. Improving transit through the Golgi in particular should enhance overall secretory flux; this process has previously been identified as a rate-limiting step for protein secretion in mammalian cells [28]. Specific upregulation of folding machinery and chaperones may also yield incremental improvements in protein production, especially for proteins with complex folds and multiple disulfide bonds. Second, identifying and modulating the regulatory elements governing the abrupt transitions between secretory states (on/off) may also confer improved productivity, regardless of protein complexity, either by reducing the mean residence time in non-secreting states or by allowing more rapid transitions between states. Third, engineering promoters controlling expression of heterologous proteins (without also addressing secretory capacity directly) should yield only modest gains in fermentation titer because this “fine-tuning” simply matches effective gene expression to the inherent secretory capacity of a cell [8]. Since the secretory biology of *P. pastoris* is similar to that found in other eukaryotic expression hosts [47], we expect these strategies to improve mechanisms of transit out of the ER via secretion should also inform approaches to enhance production of secreted heterologous proteins in other systems as well.

## Materials and Methods

### Plasmid construction

cDNA for eGFP and an aglycosylated fragment of the human Fc-region of an antibody sequence (amino acid residues 237–468) codon optimized for *P. pastoris* were designed and purchased from GeneArt (Regensburg, Germany). Each gene was inserted into a pGAPZαA plasmid (Invitrogen, Carlsbad, CA), which was modified by replacing the Zeocin resistance cassette with a kanamycin resistance cassette. Genes were cloned behind the GAPDH promoter using the restriction sites SacII and EcoRI. The resulting plasmids were named pGAPDHKαeGFP and pGAPDHKαFcAg, respectively. The plasmid pGAPDHKαFcAg was modified by site-directed mutagenesis (Quick Change II, Stratagene, Agilent Technologies, Santa Clara, CA) to recreate the glycosylation site at Asn 297 (Q→N mutation) using mutagenesis primers as follows: forward primer 5′- GCCAAGAGAAGAACAATACAACTCTACTTACAGAGTTGTTTCTG -3′ and reverse primer 5′- CAGAAACAACTCTGTAAGTAGAGTTGTATTGTTCTTCTCTTGGC -3′. The resulting plasmid was named pGAPKαFcG. The last 123 base pairs of the pGAPDH in plasmids pGAPDHKαeGFP, pGAPDHKαFcAg, and pGAPDHKαFcG were removed by digestion with BspHI and BstBI since deletion of significant portions of the 3′ region of the promoter is known to abolish promoter activity [48]. The pAOX1 gene was isolated from the pPinkHC plasmid (Invitrogen, Carlsbad, CA) using forward primer 5′-ATTAAACCATGGAGATCTAACATCCAA-3′ and reverse primer 5′-CACACTATCGATCGTTTCGAATAATTA-3′, introducing an NcoI and a ClaI site respectively. The resulting PCR fragment was digested with NcoI and ClaI and was cloned directly in front of the α-mating factor in the BspHI and BstBI digested plasmids pGAPDHKαeGFP, pGAPDHKαFcAg, and pGAPDHKαFcG to create three new plasmids named pAOX1KαeGFP, pAOX1KαFcAg, and pAOX1KαFcG, respectively.

### Strain construction

All six vectors described above were linearized in the pGAPDH gene using AvrII to target each construct to the pGAPDH locus for integration. Competent *P. pastoris* cells were prepared and transformed by electroporation according to the protocol from the pGAPZαA vector kit (Invitrogen, Carlsbad, CA). Each of the six linearized vectors was used to transform a wild-type *P. pastoris* strain (NRRL 11430) to create strains secreting a single protein of interest. The linearized pGAPDHKαeGFP was also used to transform a wild-type *P. pastoris* strain secreting glycosylated human Fc under the GAPDH promoter inserted at the TRP2 locus (a gift from GlycoFi, Inc.) to create a dual secreting eGFP/glycosylated Fc strain. Transformants were plated on YPD (10 g/L yeast extract, 20 g/L peptone, 20 g/L dextrose) plates containing 500 µg/mL G418 sulfate (Invitrogen, Carlsbad, CA). Single transformants were colony purified and used to create clonal stocks, which were kept frozen at −80°C.

### Strain cultivation

*P. pastoris* strains were streaked from frozen clonal stocks onto solid YPD media. Colonies were allowed to develop at 25°C for several days. A single colony was then used to inoculate 10 mL liquid media and the culture was then grown at 25°C with shaking at 290 rpm. Strains utilizing pGAPDH were grown in YPD medium for 18 h (OD_600_ = ∼3–6) before harvesting for further characterization. To evoke UPR, strains were grown in YPD medium for 18 h (OD_600_ = ∼3–6) before resuspension and further outgrowth in YPD containing 10 mM DTT (IBI Scientific) for 3 h. Strains utilizing pAOX1 were grown in BMGY (Buffered Glycerol Complex Medium: 100 mM potassium phosphate, pH 6.0, containing 13.4 g/L yeast nitrogen base (YNB) without amino acids, 10 g/L yeast extract, 20 g/L peptone and 10 g/L glycerol) medium for 24 h (OD_600_>10) before induction. Cells were then induced by resuspension in BMMY (Buffered Methanol Complex Medium: 100 mM potassium phosphate, pH 6.0, containing 13.4 g/L YNB without amino acids, 10 g/L yeast extract, 20 g/L peptone and 10 g/L methanol) and grown for another 18 h before harvesting for further characterization.

### Strain characterization for gene copy number

Genomic DNA was prepared from each strain using the YeaStar Genomic DNA Kit (Zymo Research, Irvine, CA) and genomic integration of the gene of interest was confirmed by PCR. Gene copy number was determined using a real-time PCR-based method as described previously [49]. Briefly, primers were designed using the PrimeTime qPCR assay design tool (IDT, Coralville, IA). Primers for copy number determination of eGFP-containing strains were as follows: forward primer 5′-GACAACCACTACCTGAGCAC-3′ and reverse primer 5′-CAGGACCATGTGATCGCG-3′. Primers for copy number determination of Fc-containing strains (either glycosylated or aglycosylated) were as follows: forward primer 5′-TGACTGTTTTGCATCAAGATTGG-3′ and reverse primer 5′-TGTGGTTCTCTTGGTTGACC-3′. Real-time quantitative PCR amplification was performed using a Roche LightCycler 480II instrument with software release 1.5.0 SP4 (Roche Diagnostics, Indianapolis, IN). Real-time PCR mixtures were prepared using the QuantiFast SYBR Green PCR kit (Qiagen, Valencia, CA) with 10 ng genomic DNA from each strain and 250 nM of each primer per assay in a total reaction volume of 25 µL. Using the thermal profile recommended in the kit, reactions were performed in LightCycler 480 96-well reaction plates in triplicate with a standard curve for each gene recorded in every plate. The amplification period was followed by a melting curve analysis with a temperature gradient of 0.1°C/s from 65° to 97°C to exclude amplification of non-specific products. The standard curve for each gene covered a copy quantity range from 1.8×10^5^ to 7.5×10^8^ copies per reaction. Calculation of copy number used the published genome size of 9.4 Mbp [14] resulting in ∼91,000 copies of the genome present in 1 ng haploid *P. pastoris* genomic DNA. Mean Ct values were plotted against their initial copy quantity and standard curves were generated by exponential regression of the plotted points. Absolute copy number for the gene of interest each strain was calculated using the mean Ct value and the corresponding gene's standard curve.

### Strain characterization for relative gene expression

Relative gene expression was determined using a reverse transcription PCR-based method as described previously [10] Briefly, total RNA was prepared from each strain using the YeaStar RNA Kit (Zymo Research, Irvine, CA). Genomic DNA was eliminated and 500 ng of RNA from each sample was reverse transcribed using the QuantiTect Reverse Transcription Kit (Qiagen, Valencia, CA). Template cDNA (corresponding to 25 ng RNA) was amplified using the QuantiFast SYBR Green PCR kit (Qiagen, Valencia, CA) with 250 nM of each primer per assay in a total reaction volume of 25 µL. The absence of genomic DNA contamination in each sample was tested by including RNA samples that had not been reverse transcribed. qPCR was performed as described above for gene copy number determination with the given primers. The relative amounts of mRNA were calculated using the Ct values and an absolute quantification of copy number from a standard curve for each gene using the actin gene as a control. Primers for actin amplification by qPCR were described previously [10].

### Strain characterization using microengraving

Microwell arrays containing 84,672 wells (each 50×50×50 µm^3^) were fabricated as reported previously using photolithography and replica molding [50]. Microwell arrays were used for microengraving with *P. pastoris* cells as previously reported [23]. Briefly, PDMS arrays were sterilized, treated, and loaded with harvested *P. pastoris* cells as cultured above. Glass slides were prepared as described [50] using 25 µg/mL goat anti-human Ig(H+L) antibody (Zymed, Invitrogen, Carlsbad, CA) as the primary antibody for Fc capture or 25 µg/mL ABfinity rabbit anti-GFP monoclonal antibody (Molecular Probes, Eugene, OR) as the primary antibody for eGFP capture. The array of microwells filled with *P. pastoris* cells was then used with the pre-treated glass slide to generate a protein microarray as described [23] during a 1 h incubation at 25°C. Following the incubation, the entire sandwich comprising the PDMS microwell array and the glass slide containing the protein microarray was submerged in sterile PBS and separated to minimize cell loss.

Following microengraving, the glass slides were washed and treated as described with a solution of either goat anti-human IgG secondary antibody (Cy5 conjugate, Jackson ImmunoResearch, West Grove, PA) at 0.5 µg/mL in PBS/Tween (0.05%) for Fc detection or rat anti-GFP secondary antibody (Alexa Fluor 647 conjugate, BioLegend, San Diego, CA) at 1 µg/mL in PBS/Tween (0.05%) for eGFP detection. Slides used to capture both eGFP and Fc from the dual secreting strain were treated with goat anti-human IgG secondary antibody (Alexa Fluor 555 conjugate) and rat anti-GFP secondary antibody (Alexa Fluor 647 conjugate) at 1 µg/mL each. Slides were imaged using a microarray scanner (GenePix 4200AL, Molecular Devices, Sunnyvale, CA) using a 635 nm or 532 nm laser and factory installed emission filters. The laser power and PMT gain were set to maximize the linear range of detection in each experiment. The fluorescence intensity for each individual spot on the engraved protein microarray was converted to a quantity of protein using a standard curve. The standard curve was obtained by constructing a protein array using known quantities of Fc or eGFP (50, 25, 5, 1, 0.5 and 0.1 ng/mL) diluted in appropriate media (either YPD or BMMY) and spotted on a glass slide as treated above. The slide was incubated for 1 h, then developed and imaged as described above in parallel with microengraved protein arrays generated on the same day. Background-corrected fluorescence values were plotted against concentration to determine the linear range of the microengraving assay.

### In-well imaging cytometry

Phase contrast and fluorescence images of the cell-loaded PDMS microarray were acquired using AxioVision software (v4.7.2, Carl Zeiss MicroImaging, Thornwood, NY) and an automated inverted microscope (AxioObserver Z1, Carl Zeiss, MicroImaging, Thornwood, NY) equipped with a Hamamatsu EM-CCD camera.

### Data processing and statistical analysis

Phase contrast and fluorescence images of the cell-loaded PDMS microarray were analyzed for identification of the number of cells in each well using custom software. Images of the printed microarrays were analyzed using GenePix Pro 6.0 (Molecular Devices, Sunnyvale, CA). The background intensity for each array was determined from the median of all values measured in regions between individual spots of the array. Spots in the array were identified as positive when the signal-to-noise ratio was greater than 2–that is, when the spot intensity was greater than the sum of the background intensity for the array plus two standard deviations of the values used to calculate the background intensity. Multidimensional data were correlated using a custom script. All subsequent data filtering and analysis was performed using Microsoft Excel, MatLab, or GraphPad (statistical analysis). Heatmap representations of population distributions were generated using GenePattern [51].

### Gamma distribution fitting of population distributions of secretion

Population distribution histograms were fitted to the gamma distribution (**Eq. 1**) using a constrained optimization function written in MATLAB. The parameters *a*, *b*, and λ, a scaling factor for the histogram data, were constrained (10^−2^<*a*<10^2^; 10^−2^<*b*<10^2^; 10^−5^<λ<10^2^) and then optimized to maximize the R^2^ value of the fit between the scaled histogram data and the gamma distribution determined by *a* and *b*.

### Secretory pathway modeling

The secretion performance of individual cells was modeled using a system of mass-balance ordinary differential equations (ODEs, **Eqs. 2** **–** **4**), which were solved using an ODE solver in MATLAB. Cells were assumed to start with no protein product in any of the three compartments defined above, those being the ER, proteasome, or secretory pathway. To account for the epigenetic diversity within a population of cells, t_sec_ (1/k_sec_) and t_ERAD_ (1/k_ERAD_) were sampled from a Gaussian distribution, with a standard deviation between approximately 7 to 17 percent of the mean. The median value of t_sec_ was initially chosen as 80 min, based on data from protein secretion in mammalian cells [28], and the median values of t_sec_ and t_ERAD_ were varied from 60 to 140 min. The initial value for k_exp_ was chosen as 1 molecule/s, which corresponds to a rate of ∼1.4 ng*mL^−1^*h^−1^ for GFP in our microwells. The ODEs were solved over 20,000 s to ensure the system reached steady-state—that is, when [ER] only changed by 0.002% between the penultimate and ultimate steps of the ODE solver, at which point the secretion rate and amount of protein in the ER were recorded. This process was repeated 5,000 to 10,000 times to generate representative model populations of cells.

## Supporting Information

Figure S1 **Plot of relative gene expression for strains listed in** **Table 1** **using pGAPDH against the median single-cell rate of protein secretion for each strain as determined by microengraving.** Each median value is an average of at least three replicate microengraving measurements per strain. Data were fit by linear regression (R^2^ = 0.83).
(TIF)

Figure S2 **Composite fluorescent micrographs acquired by confocal microscopy of** ***P. pastoris*** **strains containing a single-gene copy of eGFP (A) with an upstream α-mating factor signal sequence (for trafficking through the secretory pathway) or (B) without a signal sequence (for intracellular expression). Cells were isolated in microwells (dark edges).** Magnification was 63×.
(TIF)

Figure S3 (A, B) Distributions of single-cell rates of glycosylated Fc fragment secretion during either shake-flask cultivation or fed-batch fermentation (3L). Distributions are shown for the point of best induction during either cultivation and median rates of secretion are similar for each using either the (A) pGAPDH or (B) pAOX1 promoter. (C, D) Scatter plot of time-dependent cell growth (blue diamonds) and product titer (corrected by wet cell mass, red squares) for reactors producing glycosylated Fc fragment using either the (C) pGAPDH or (D) pAOX1 promoter. Black dashed line shows the point of induction for the cultivation.
(TIF)

File S1 Single-cell data from representative experiments with each eGFP strain listed in Table 1. Included are the median fluorescent intensities for secreted GFP measured by microengraving, compensated median fluorescent intensities for intracellular GFP measured by in-well cytometry, and the calculated rate of secretion for eGFP-secreting single cells based on calibration curves collected with each experiment. The data are divided into two groups: single cells exhibiting secretion of GFP (MFI> background+2SD) and single cells with secretion below the limit of detection. The cut-off values for each representative dataset are indicated.
(XLSX)
