# Supplementary figures and images for: Systematic Single-Cell Analysis of Pichia pastoris Reveals Secretory Capacity Limits Productivity
Source: PLoS One. 2012 Jun 7;7(6):e37915. doi: 10.1371/journal.pone.0037915 (PMC3369916; doi:10.1371/journal.pone.0037915)

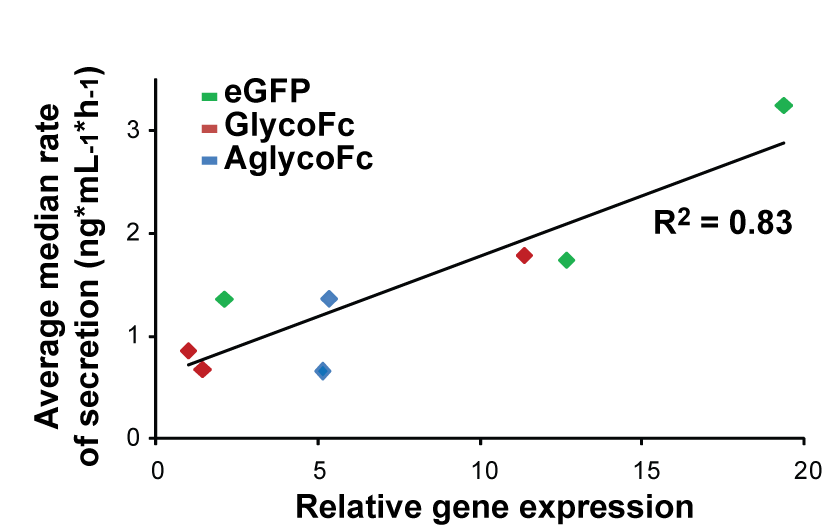

Supplement: Figure S1 — Plot of relative gene expression for strains listed in Table 1 using pGAPDH against the median single-cell rate of protein secretion for each strain as determined by microengraving. Each median value is an average of at least three replicate microengraving measurements per strain. Data were fit by linear regression (R2 = 0.83). (TIF) [file pone.0037915.s001.tif]

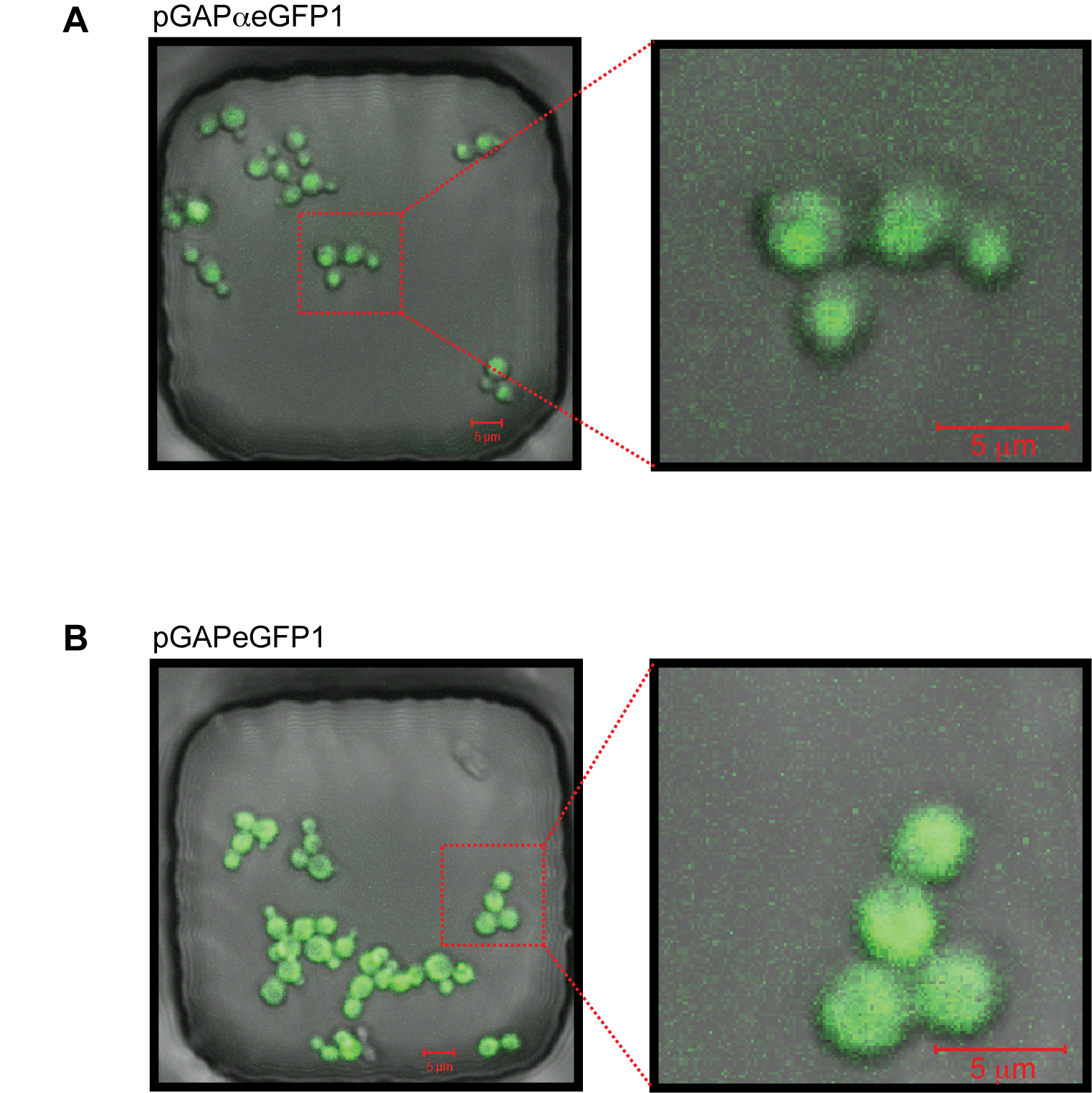

Supplement: Figure S2 — Composite fluorescent micrographs acquired by confocal microscopy of P. pastoris strains containing a single-gene copy of eGFP (A) with an upstream α-mating factor signal sequence (for trafficking through the secretory pathway) or (B) without a signal sequence (for intracellular expression). Cells were isolated in microwells (dark edges). Magnification was 63×. (TIF) [file pone.0037915.s002.tif]

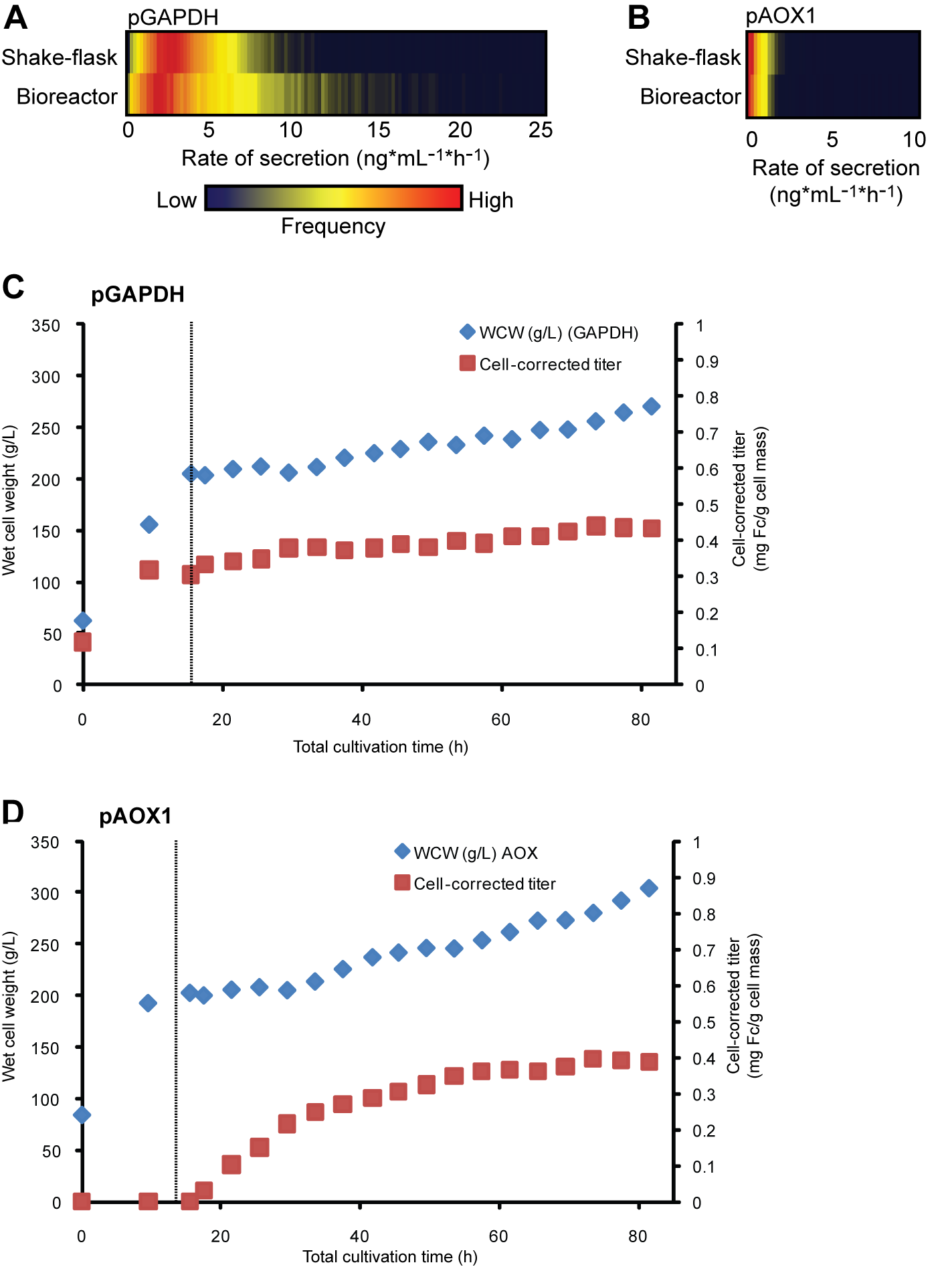

Supplement: Figure S3 — (A, B) Distributions of single-cell rates of glycosylated Fc fragment secretion during either shake-flask cultivation or fed-batch fermentation (3L). Distributions are shown for the point of best induction during either cultivation and median rates of secretion are similar for each using either the (A) pGAPDH or (B) pAOX1 promoter. (C, D) Scatter plot of time-dependent cell growth (blue diamonds) and product titer (corrected by wet cell mass, red squares) for reactors producing glycosylated Fc fragment using either the (C) pGAPDH or (D) pAOX1 promoter. Black dashed line shows the point of induction for the cultivation. (TIF) [file pone.0037915.s003.tif]
